# Urinary drug metabolite testing in chronic heart failure patients indicates high levels of adherence with life‐prolonging therapies

**DOI:** 10.1002/ehf2.13284

**Published:** 2021-03-11

**Authors:** Mark Sweeney, Graham D. Cole, Punam Pabari, Savvas Hadjiphilippou, Upasana Tayal, Jamil Mayet, Neil Chapman, Carla M. Plymen

**Affiliations:** ^1^ Imperial College Healthcare NHS Trust London UK; ^2^ Imperial College London London UK

**Keywords:** Heart failure, ACE inhibitor, Beta‐blocker, Aldosterone antagonist, Adherence, Urinary testing

## Abstract

**Aims:**

Despite medical therapy for heart failure (HF) having proven benefits of improving quality of life and survival, many patients remain under‐treated. This may be due to a combination of under‐prescription by medical professionals and poor adherence from patients. In HF, as with many other chronic diseases, adherence to medication can deteriorate over time particularly when symptoms are well controlled. Therefore, detecting and addressing non‐adherence has a crucial role in the management of HF. Significant flaws and inaccuracies exist in the methods currently used to assess adherence such as patient reporting, pill counts, and pharmacy fill records. We aim to use high‐performance liquid chromatography–tandem mass spectrometry (HPLC‐MS) to detect metabolites of HF medications in the urine samples of chronic HF patients.

**Methods and results:**

Urine samples were collected from 35 patients in a specialist HF clinic. Patients were included if they had an ejection fraction <45% and were taking at least two disease‐modifying HF medications. They were excluded if they had been admitted to hospital for HF in the 3 months preceding clinic attendance. These samples were sent for HPLC‐MS and tested for all HF medications prescribed for that patient. A high rate of complete adherence of 89% was detected in these patients, with 94% being partially adherent (at least one HF medication detected) to therapy (at least one HF medication detected). This analysis also highlighted that mineralocorticoid antagonists represent both the most under‐prescribed (67%) and poorly adhered (75%) medication class.

**Conclusions:**

This analysis revealed a surprisingly high level of adherence to disease‐modifying therapy in chronic HF patients and highlights that most of our ‘total’ under‐treatment is likely to be from a failure to prescribe rather than a failure to adhere. Testing for metabolites of disease‐modifying HF drugs in urine using HPLC‐MS is feasible and is a useful adjunct to a specialist HF service. At present, the distinction between treatment failure and failure to take treatment is not always clear, which is important because the investigation and potential solutions are different. The former needs initiation of additional therapies and consideration of additional diagnoses, whereas the latter requires strategies to understand reasons underlying poor adherence and collaborative working to improve this: the wrong strategy will be ineffective.

## Background

Medical therapy for heart failure (HF) is known to improve symptoms and prolong survival. However, as with other chronic diseases,^1^, ^2^, ^3^ adherence to therapy is variable, can be challenging to uncover,^4^ and is associated with adverse prognosis in HF.^5^ Recent studies have demonstrated an adherence rate to disease‐modifying HF treatment of 89% in patients recently discharged from hospital following a HF admission.^6^ However, in chronic HF patients who may be months or years after their initial diagnosis and may be currently free from symptoms, the motivation to continue taking prognostic HF therapies may diminish. Maintaining high levels of adherence in these long‐term HF patients will ensure the ongoing benefits from these treatments in reducing hospitalization, maintaining quality of life, and improving survival.

Methods used to assess adherence at present, such as self‐reporting, pill counts, pharmacy refill records, and electronic medication monitoring, have potential pitfalls including inaccuracies in self‐reporting and collected but unused medication. This limits the ability to assess the true prevalence of non‐adherence to medical therapy; studies indicate that adherence ranges from 33%^7^ to 76%,^8^ and the adherence rates within the same study can vary widely depending on the method used to assess this.^7^ If non‐adherence remains undetected, the underlying causes cannot be addressed to the detriment of patient care.

Given the importance of establishing and measuring adherence in chronic HF patients, we aimed to assess adherence in a real‐world group of chronic HF patients who did not have any recent admissions to hospital with HF.

## Methods

Patients with a diagnosis of HF attending a follow‐up appointment in a single specialist HF clinic were recruited over a period of 3 months. Patients were included in the study if they had an ejection fraction <45% and were prescribed with at least two disease‐modifying HF medications. Those with an HF admission in the preceding 3 months were excluded. All patients were fully informed on the day of clinic that drug metabolites were being measured and no patients declined to provide a sample when invited to take part.

High‐performance liquid chromatography–tandem mass spectrometry (HPLC‐MS) was used to detect urinary drug metabolites for all disease‐modifying HF drugs prescribed to each patient. Metabolites for all drugs tested remain present in the urine for 24 h following ingestion,^6^ meaning that any medication taken on the day of the appointment will be detectable. We did not test for loop diuretic therapy as the duration for detection of these drugs is shorter and more variable and adherence to loop diuretic therapy on the day of a clinic appointment may not be representative of true adherence.

Continuous data are presented as mean and standard deviation where data are normally distributed and median and inter‐quartile range when the distribution is non‐normal. Categorical data are presented as number and percentage of the total population.

## Results

Thirty‐five patients who met the inclusion criteria were included in the analysis. The characteristics of those included in the study are summarized in *Table* *1*. There was an equal split of ischaemic (49%) and non‐ischaemic (51%) aetiologies represented. A wide spectrum NYHA class was included in the study population; class I (23%), II (51%), and III (26%). There were no admissions to hospital within the preceding 6 months.

**Table 1:** Characteristics of patients included in the study and individual characteristic of non‐adherent patients

Patient characteristics	All patients (*n* = 35)	Non‐adherent patients (*n* = 4)			
		Patient 1	Patient 2	Patient 3	Patient 4
Female, *n* (%)	9 (26)	Male	Male	Male	Male
Age (years)	66 (56–73)	>65	>65	>65	<65
**Aetiology**					
Ischaemic, *n* (%)	17 (49)	Ischaemic	Ischaemic	Non‐ischaemic	Ischaemic
Non‐ischaemic, *n* (%)	18 (51)				
**Symptoms**					
NYHA class					
I, *n* (%)	8 (23)	II	II	I	II
II, *n* (%)	18 (51)				
III, *n* (%)	9 (26)				
MLHFQ	17 (0–41)	33	15	13	20
**Physiological parameters**					
LVEF (%)	32 (25–37)	24	29	19	25
SBP (mmHg)	122 (135–110)	126	91	135	149
HR (b.p.m.)	65 (75–57)	85	92	64	59
BNP (ng/L)	381 (76–514)	392	392	59	76
Creatinine (μmol/L)	98 (82–139)	186	210	101	74
**Prognostic therapy**					
ACE/ARB/ARNI	35 (100)	Candesartan ✓	Candesartan ✓	Ramipril ✕	Ramipril ✕
ACE‐I, *n* (%)	22 (63)				
ARB, *n* (%)	9 (26)				
ARNI, *n* (%)	4 (11)				
Beta‐blocker, *n* (%)	34 (97)	Bisoprolol ✓	Bisoprolol ✕	Bisoprolol ✕	Bisoprolol ✕
MRA, *n* (%)	15 (43)	Spironolactone ✕	Spironolactone ✕	Spironolactone ✕	Eplerenone ✕
CRT, *n* (%)	13 (37)	CRT‐D	CRT‐D	CRT‐D	ICD
ICD, *n* (%)	17 (49)				

ACE‐I, angiotensin‐converting enzyme inhibitor; ARB, angiotensin receptor blocker; ARNI, angiotensin receptor blocker–neprilysin inhibitor; BNP, brain natriuretic peptide; CRT, cardiac resynchronization therapy; CRT‐D, cardiac resynchronization therapy defibrillator; HR, heart rate; ICD, implantable cardioverter defibrillator; LVEF, left ventricular ejection fraction; MLHFQ, Minnesota Living with Heart Failure Questionnaire; MRA, mineralocorticoid receptor antagonist; NYHA, New York Heart Association; SBP, systolic blood pressure.

Continuous data presented as mean (standard deviation) when normally distributed and median (inter‐quartile range) when non‐normally distributed. Categorical data presented as *n* (% of total population).

All patients were prescribed with one of angiotensin‐converting enzyme inhibitor (63%), angiotensin receptor blocker (26%), or angiotensin receptor blocker–neprilysin inhibitor (11%). Beta‐blockers were prescribed to 34 patients (97%), and 15 patients (45%) had been prescribed with mineralocorticoid receptor antagonists (MRAs). The rates of prescription of the three main classes of HF medication in this study mirrored those found in the National Institute for Cardiovascular Outcomes Research HF audit 2019.^9^

**Figure 1:**
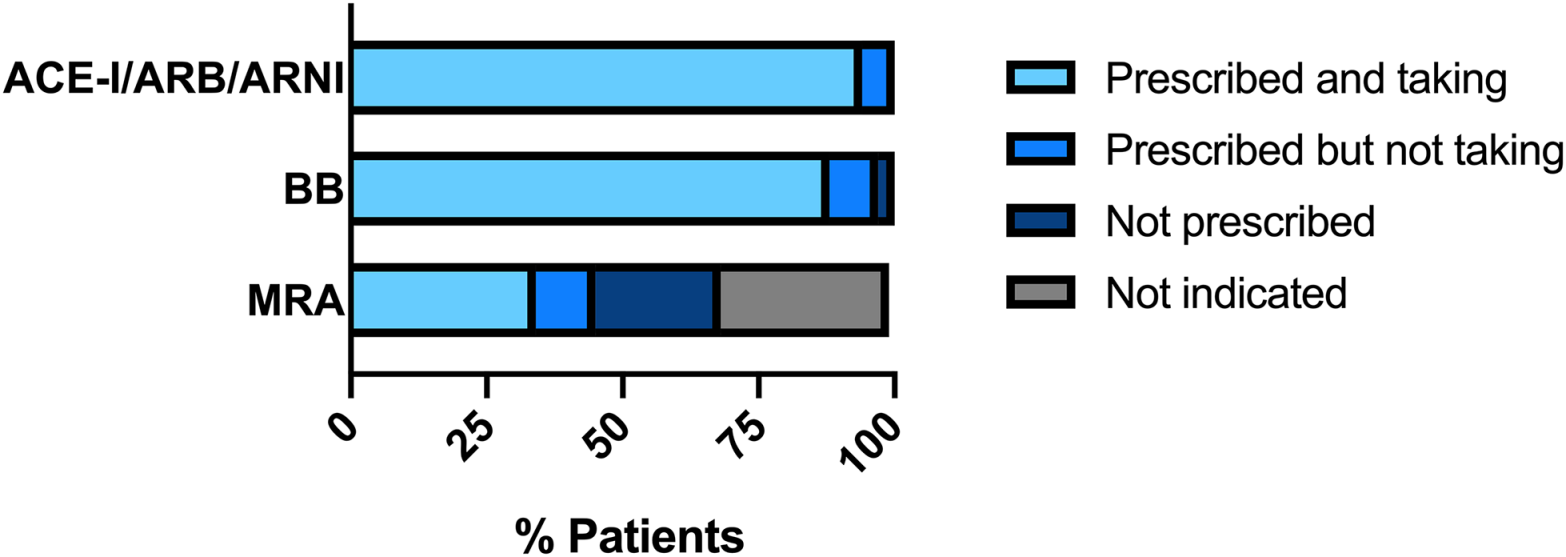
Proportion of patients adherent to each class of prescribed heart failure medication compared with the proportion of non‐adherent patients, patients not prescribed this medication class, or patients in which the medication class is contraindicated. ACE‐I, angiotensin‐converting enzyme inhibitor; ARB, angiotensin receptor blocker; ARNI, angiotensin receptor blocker–neprilysin inhibitor; BB, beta‐blocker; MRA, mineralocorticoid receptor antagonist.

Thirty‐one (89%) patients had urinary metabolite results indicating that they were taking all of their prescribed disease‐modifying HF medications. Thirty‐three patients (94%) were partially adherent to at least one HF medication with only two patients (6%) being completely non‐adherent to any HF medication. (*Figure* *1*). Non‐adherence to MRA was most common, omitted by all four non‐adherent patients representing a relatively low adherence rate (75%) compared with beta‐blockers (91%), inhibitors of the renin–angiotensin pathway (94%), and sacubitril/valsartan (100%).

## Conclusions

These findings indicate that in the setting of a specialist multidisciplinary HF clinic, which includes an education programme delivered via specialist nurses, adherence to HF therapy can be maintained in chronic HF patients at levels equivalent to recently discharged patients.^6^ However, as is seen nationally, despite 24 patients being eligible for MRA, a high proportion were not receiving this drug class (50%). This under‐treatment is contributed to by both non‐prescription (33%) and non‐adherence (17%). MRAs are often prescribed later during the patient journey, and reinforcing the importance of medication adherence at this stage may be an important opportunity to improve adherence and therefore quality of life and mortality.

Accurately differentiating between *treatment failure* compared with *failure to treat* or *failure to take treatment* allows bespoke solutions to be devised. Treatment failure may need initiation of additional therapies or consideration of additional diagnoses, whereas the failure to take treatment requires strategies to understand reasons underlying poor adherence and collaborative working to improve this: the wrong strategy will be ineffective.

The biggest limitation of this study is the small sample size of 35 patients; however, it provides an effective proof of concept. Additionally, in the real‐world setting of a specialist HF outpatient clinic, patients are already invested in their care and adherence is therefore likely to be higher than in other settings.

In conclusion, in this group of patients who are managed in a contemporary multidisciplinary HF service, there were high levels of adherence to therapy. If true on a larger scale, the implications for prescribers are significant: most of our ‘total’ under‐treatment is likely to be from a failure to prescribe rather than a failure to adhere. Urinary drug metabolite testing may be a useful adjunct to an HF service and provides an effective strategy to assess adherence in patients who are not responding to treatment. It may also have a role in clinical trials, to evaluate the effect of interventions to improve adherence and to audit the effectiveness of HF services.

## Conflict of interest

None declared.

## Funding

This study was supported by a grant from the Nissan Cardiology Charitable Fund and the NIHR Imperial Biomedical Research Centre (BRC). M.S is supported by the Wellcome Trust (220573/Z/20/Z).
